# Upcoming evidence in clinical practice of two-stage revision arthroplasty for prosthetic joint infection

**DOI:** 10.1186/s10195-024-00767-1

**Published:** 2024-05-18

**Authors:** Tiziana Ascione, Giovanni Balato, Pasquale Pagliano

**Affiliations:** 1 Service of Infectious Diseases, Cardarelli Hospital, Via A. Cardarelli 9, 80131 Naples, Italy; 2 Department of Public Health, Orthopedic Unit, “Federico II” University, Naples, Italy; 3 Unit of Infectious Diseases, Department of Medicine, Surgery and Dentistry, “Scuola Medica Salernitana”, University of Salerno, Baronissi, Italy; 4 Clinica Malattie Infettive, AOU San Giovanni di Dio e Ruggi d’Aragona, Salerno, Italy

**Keywords:** Prosthetic joint infection, Two-stage, Reimplantation, Synovial fluid examination, D-dimer, Scoring system

## Abstract

Total joint arthroplasty is the recommended treatment for patients with end-stage osteoarthritis, as it reduces disability and pain and restores joint function. However, prosthetic joint infection is a serious complication of this procedure, with the two-stage exchange being the most common treatment method. While there is consensus on diagnosing prosthetic joint infection, there is a lack of agreement on the parameters that can guide the surgeon in performing definitive reimplantation in a two-stage procedure. One approach that has been suggested to improve the accuracy of microbiologic investigations before definitive reimplantation is to observe a holiday period from antibiotic therapy to improve the accuracy of cultures from periprosthetic tissues, but these cultures report some degree of aspecificity. Therefore, several pieces of evidence highlight that performing reimplantation using continuous antibiotic therapy should be considered a safe and effective approach, leading to higher cure rates and a shorter period of disability. Dosage of C-reactive protein (CRP), erythrocyte sedimentation rate (ERS) and D-dimer are helpful in diagnosing prosthetic joint infection, but only D-dimer has shown sufficient accuracy in predicting the risk of infection recurrence after a two-stage procedure. Synovial fluid analysis before reimplantation has been shown to be the most accurate in predicting recurrence, and new cutoff values for leukocyte count and neutrophil percentage have shown a useful predictive rule to identify patients at risk of unfavourable outcome. A new scoring system based on a numerical score calculated from the beta coefficient derived through multivariate analysis of D-dimer levels, synovial fluid leukocytes and relative neutrophils percentage has demonstrated high accuracy when it comes to guiding the second step of two-stage procedure. In conclusion, reimplantation may be a suitable option for patients who are on continuous therapy without local symptoms, and with CRP and ERS within the normal range, with low synovial fluid leukocytes (< 952/mL) and a low relative neutrophil percentage (< 52%) and D-dimer below 1100 µg/mL. A numerical score derived from analysing these three parameters can serve as a valuable tool in determining the feasibility of reimplantation in these patients.

## Introduction

Total joint arthroplasty is a commonly performed procedure to alleviate pain and improve joint function of patients with end-stage osteoarthritis. While this procedure is highly standardized and affordable for many patients, several complications can lead to implant failure and may necessitate revision surgery or other interventions [1].

Prosthetic joint infection (PJI) is a serious complication of total joint arthroplasty. Data obtained from the US Department of Veterans Affairs on a sample of around 80,000 patients undergoing primary total knee arthroplasty identified a percentage of patients with PJI approaching 2%, with the highest number of PJI cases diagnosed within 24 months from primary knee arthroplasty [2]. Further data obtained by a meta-analysis including articles investigating the incidence of PJI after primary hip arthroplasty highlight an incidence approaching 1%, with the highest number of cases among people aged ≥ 70 years and a great difference in terms of incidence among different countries [3].

The management of chronic PJI is indeed challenging, as bacteria embedded in biofilm do not allow for definitive microbiological cure outside a surgical approach consisting of infected prosthesis removal. Two-stage implant replacement is considered the most common surgical approach, as it allows the healing of the periprosthetic tissues infection during the time elapsing from infected implant removal and new prosthesis implantation and minimizes the risk of PJI recurrence [4, 5].

Ideally, any procedure effective in assessing the definitive cure of the periprosthetic tissue infection prior to reimplant would be useful to report the highest success rate after two-stage replacement. In any case, no consensus has been obtained on examinations to be performed prior to reimplantation, as the same criteria applied at the time of PJI diagnosis do not have sufficient specificity and sensibility to exclude infection persistence with 100% accuracy [6].

In this narrative review, we provide an outlook on the current concepts in two-stage exchange management of chronic PJI, including the optimal antibiotic treatment regimen and the evaluation of all parameters guiding clinicians to define the ideal timing of reimplantation after spacer placement.

## Unanswered questions in two-stage revision: criteria to be adopted to exclude PJI persistence at the time of reimplantation

Applying MusculoSkeletal Infection Society (MSIS)-18 criteria is a widely accepted method for diagnosing PJI. However, assessing PJI cure during the two-stage replacement process can be difficult due to the absence of a definitive parameter that reliably predicts the eradication of the infection from the periprosthetic tissues before reimplantation of the prosthetic joint [6]. For this purpose, several issues have been raised to lower the risk of PJI recurrence: (i) holiday period versus continuous antibiotic therapy before reimplantation, (ii) the usefulness of microbiology at reimplantation, (iii) the usefulness of serum and plasma biomarkers at the time of reimplantation and (iv) the usefulness of synovial fluid analysis and identification of the cutoff for leukocyte and relative neutrophil percentages to guide reimplantation.

## Holiday period versus continuous therapy and microbiology at reimplantation

The impact of a holiday period from antibiotic therapy prior to definitive reimplantation on sensitivity and accuracy of cultures obtained from preoperative and intraoperative synovial fluid aspirates before reimplantation and from periprosthetic tissue cultures at the time of reimplant is indeed a topic that deserves some consideration. In a retrospective study on 267 cases of PJI undergoing two-stage exchange, the authors found a 24% recurrence rate. Additionally, they noted a twofold increase in the infection recurrence rate among cases that had positive cultures at the time of reimplantation. This study highlights that the bacteria retrieved at reimplantation were the same as the initial infecting organism in only 6 (18%) cases, and only 11 cases (33%) reported more than one positive specimen, bringing into question the effectiveness and usefulness of the data obtained by the microbiologic investigations performed after antibiotic therapy discontinuation. However, the lack of specific details about the antibiotic protocol and patient characteristics in the study limits our ability to draw definitive conclusions about the effectiveness of the holiday period from antibiotic therapy [8]. The value of microbiological data obtained at reimplantation in predicting recurrence is brought into question by another study reviewing the medical charts of 84 patients who underwent implant revision after an antibiotic holiday period of 4 weeks. In fact, only 2 of 25 cases with bacterial growth on spacers (2 or more positive samples in 10 and 1 positive sample in 15) experienced infection recurrence, and there was no statistical difference in the recurrence rate compared with those that did not report bacterial growth on spacer. Additionally, 18 cases reported the growth of bacteria different than those found at the time of infected implant removal, raising questions about the significance of microbiologic investigations during reimplantation [9].

A meta-analysis investigating the risk of complications in patients with positive cultures at the time of reimplantation confirmed the hypothesis that patients with one or more positive cultures are at increased risk of procedure failure, but some issues need to be underscored about the relationship occurring between cultures, protocol of antibiotic treatment and cure rate. In fact, subgroup analysis did find a slightly higher positive culture rate among those observing a holiday period from antibiotic therapy but found that the association between positive culture at reimplantation and unfavourable outcomes was consistent regardless of the antibiotic protocol used. Interestingly, the study also found that patients with a positive culture at reimplantation receiving continuous antibiotic therapy reported a lower recurrence rate [10, 11]. This can suggest that adopting continuous antibiotic therapy probably reduces the risk of complications in patients with positive cultures at reimplantation.

Undergoing reimplantation with a holiday period does not provide any significant advantage in terms of culture accuracy at the time of definitive reimplantation based on these data and on some evaluations. First, continuous antibiotic therapy can offer an advantage in terms of cure rate and reduces time of disability, as it shortens the time between the two steps of the procedure by avoiding the holiday period. Indeed, results of a retrospective study investigating 101 patients undergoing two-stage exchange underscore that a longer period between the infected prosthesis explant and definitive reimplantation is associated with an increase in the rate of readmission and failure [12]. Similar data are reported in another retrospective study where a spacer retention period > 11 weeks was associated with an unfavourable outcome [13]. Second, avoiding a holiday antibiotic period shortens the spacer persistence period and reduces the risk of reinfection as bacteria within biofilms on the spacer surface can migrate to nearby tissue when antibiotic therapy is interrupted [14, 15]. Third, administering continuous antibiotic therapy until spacer removal, debridement and reimplantation could theoretically reduce the risk of PJI recurrence, particularly when a partial infection eradication was obtained [16]. The advantage obtained by two different schedules of treatment which did or did not consider antibiotic therapy withdrawal before definitive reimplantation is a matter of debate. Ascione et al. investigated two large cohorts undergoing two-stage replacement after PJI observing 2 weeks of holiday period from antibiotic treatment before reimplantation or receiving continuous antibiotic therapy until reimplantation. Adopting continuous therapy was an independent factor associated with favourable outcome [odds ratio (OR), 3.32; 95% confidence interval (CI), 1.3–8.44; *P* = 0.02], as assessed by multivariate analysis. Additionally, immunocompromised patients showed the greatest benefit in terms of cure rate with the schedule considering continuous therapy. Table 1 reports the results of the main study investigating the usefulness of an antibiotic holiday period before definitive reimplantation [8, 16–23].

**Table 1:** Studies investigating the outcome of two-stage exchange in patients with prosthetic joint infection

Author (reference)	Patients	Procedure	Joint	Percentage with favourable outcome (%)
Corrò S [17]	108	Holiday period	Hip and knee	78
Saade A [18]	50	Holiday period	Hip and knee	92
Ascione T* [16]	65	Holiday period	Hip and knee	79
Ascione T* [16]	104	Continuous therapy	Hip and knee	91
Akgün D [19]	163	Continuous therapy	Hip and knee	83
Carrega G [20]	102	Holiday period	Hip and knee	85
Hart WS [21]	48	Not reported	Knee	88
Cabo J [22]	55	Holiday period	Hip and knee	88
Nelson CL [23]	36	Holiday period	Hip and knee	67
Tan [8]	259	Holiday period	Hip and knee	75

* Data are derived by the same study

## Serum and plasma biomarkers at the time of reimplantation

The use of serum biomarkers such as C-reactive protein (CRP), erythrocyte sedimentation rate (ERS) and D-dimer in distinguishing PJI from aseptic prosthetic implant loosening is well-established by MSIS-18 criteria. However, their role in guiding reimplantation remains a topic of debate [24]. Several studies report that CRP and ESR are not unequivocal biomarkers for assessing microbiologic eradication after implant removal in individuals undergoing a two-stage exchange procedure. For example, a study evaluating changes in CRP and ESR levels prior to infected prosthesis explantation and periprosthetic tissue debridement and after 6 weeks of targeted antibiotic therapy found that the decrease in CRP and ESR levels did not consistently predict infection recurrence [25]. In contrast to this study, another study examining the prognostic value of ESR and CRP prior to the second stage in 198 patients affected with PJI of the knee indicated a predictive role of both biomarkers in identifying the patients with the highest risk of recurrence. However, it was observed that, even when these biomarkers are within the normal range, there is a still significant recurrence rate of around 15%. The results of this study emphasize the importance of not solely relying on individual biomarkers such as CRP and ESR but rather incorporating a multidimensional assessment that considers various clinical, laboratory and radiological parameters to provide a more accurate risk assessment and guide treatment decisions for patients with PJI [26].

D-dimer is a product of fibrinolysis, whose levels can be influenced by a reparative and coagulative process, or by inflammatory cascade activation. Monitoring D-dimer levels can be valuable in identifying septic complications in patients beyond the postoperative period and in patients without a hypercoagulative status because D-dimer levels can be affected both by thrombus formation and thrombolytic activity. Indeed, Shahi et al. have demonstrated that D-dimer reported high sensibility and specificity in supporting the diagnosis of chronic PJI [27].

Moreover, further investigations have validated the role of serum D-dimer in ruling out a prosthetic infection, adopting a cutoff value higher than proposed by Shahi et al. [24, 27–29]. In a study involving 125 patients with aseptic loosening or PJI of a knee implant, D-dimer dosage at a different cutoff was found to be more accurate than ESR and CRP in diagnosing PJI [24]. Additionally, there is limited research exploring the predictive value of serum D-dimer in identifying the patients at the highest risk of recurrence during the two-stage exchange procedure [30]. Tarabichi et al. demonstrated that higher level of D-dimer, but not CRP or ESR levels, were associated with infection recurrence in a study enrolling patients undergoing reimplantation after a 2-week holiday period [31]. Conversely, Pannu et al. concluded that D-dimer alone had poor accuracy in predicting reinfection following reimplantation [32]. The conflicting results on the value of D-dimer in suggesting a higher risk of PJI recurrence can be explained by a study investigating the dynamics of D-dimer in a cohort of 30 patients undergoing two-stage exchange. This study revealed an increase in plasma D-dimer levels from pre-explantation to pre-reimplantation, regardless of the two-stage procedure outcome. These results raise doubts about the value of this marker in guiding treatment decisions in two-stage exchange procedure but emphasize the importance of contextualizing the effective value of D-dimer within a broader clinical framework [33].

Indeed, a meta-analysis analysing 47 randomized controlled trials and comparative observational studies further supports the limited prognostic value of serum or plasma biomarkers in identifying patients with a significant risk of infection recurrence before definitive reimplantation [30]. The findings of this analysis suggest that no single serum biomarker evaluated before the second stage of the two-stage revision has sufficient specificity and sensibility to predict infection cure with a high sensibility and specificity.

The summary of the main studies investigating the role of serum and plasma biomarkers in predicting infection recurrence is provided in Table 2.

**Table 2:** Summary of the main studies investigating the role of serum and plasma biomarkers in predicting infection recurrence

Author (reference)	Patients (number)	Joint	Biomarker	Predictive role
Stambough JB [25]	300	Hip and knee	ESR and CRP	No
Klemt C [26]	198	Hip and knee	ESR and CRP	Yes (when both elevated)
Shahi A [27]	245	Hip and knee	D-dimer	Yes (higher than both CRP and ESR)
Li R [28]	565	Hip and knee	Fibrinogen	Yes
Li R [28]	565	Hip and knee	D-dimer	Low value
Li R [28]	565	Hip and knee	ESR and CRP	Yes
Tarabichi S [31]	114	Hip and knee	D-dimer	Yes (higher than both CRP and ESR)
Pannu TS [32]	53	Hip and knee	D-dimer	Yes (higher when combined with ESR/CRP)

## Synovial fluid analysis at reimplantation

Synovial fluid investigations, which encompass procedures such as microbiological cultures and leukocytes count and determination of the relative neutrophil percentage, play a crucial role in evaluating the eradication of infection at the time of definitive reimplantation. Among cases whose diagnosis remains uncertain, an additional synovial fluid a-defensin test can be proposed.

Studies assessing the role of microbiologic investigations on synovial fluid face limitations due to differences in terms of diagnostic and therapeutic protocols [34]. While microbiologic investigations on synovial fluid report a favourable predictive value in diagnosing PJI, their role after implant removal should be considered in light of several aspects. In fact, sensibility and specificity of synovial fluid cultures can be affected by several factors, including the systemic antibiotic therapy, the local antibiotic release from spacer or the presence of bacteria in the surrounding periprosthetic tissues in a non-planktonic form [35]. A retrospective study on 50 patients undergoing the two-stage process due to PJI revealed infection recurrence in 5 patients whose synovial fluid cultures were negative before definitive reimplantation [9]. Similar findings have been observed in other investigations, demonstrating that sensitivity and specificity of synovial fluid culture before or during definitive reimplantation can be suboptimal [36–38]. All these investigations make the result of synovial fluid cultures alone at the time of reimplantation of low accuracy when it comes to predicting PJI recurrence.

In addition to microbiologic cultures, investigating synovial fluid for leukocyte count and relative neutrophil percentage or a-defensin can provide other helpful information. As reported for serum or plasma biomarkers, current cutoff values for synovial fluid leukocytes count and relative neutrophil percentage currently used to diagnose PJI are not accurate enough to rule out persistent infection at the time of reimplantation [39]. Additionally, Bian et al. [40] have reported extreme variability in the sensitivities and specificities of synovial fluid leukocytes count and neutrophil percentage, when they were used to identify persistent infections before definitive reimplantation. Newman et al. [41] and Zmistowski et al. [42] have proposed new cutoff values for synovial fluid leukocytes counts and neutrophil percentage to detect patients with persistent infections. Starting from the hypothesis that synovial fluid examination can predict patients at high risk of recurrence, Ascione et al. reported that only 18% of patients experiencing recurrence had leukocyte counts or neutrophil percentages above the limits required as established by the International Consensus Meeting (ICM) 2018 for diagnosing PJI, despite having CRP and ESR within normal range or downloading [43]. Furthermore, analysing synovial fluid examination results before reimplantation in a cohort of 82 patients receiving continuous antibiotic therapy until reimplanatation and undergoing two-stage exchange without clinical or laboratory findings suggesting an ongoing infection, Ascione et al. indicated that synovial leukocytes count over 934 cells/mL or neutrophil percentage over 52% were associated with a higher risk of persistent or recurrent PJI [43].

Different cutoff values have been proposed [44, 45], but their role should be evaluated in light of the different protocols adopted, particularly in patients who did not undergo an antibiotic holiday period before reimplantation. Zmistowski et al. [42] determined that a leukocyte count of 640 cells/mL and a neutrophil percentage of 56% were excellent thresholds for diagnosing persistent infections, while Kusuma et al. [44] reported a synovial fluid leukocyte count of 1102 cells/mL and a neutrophil percentage of 71.5% as thresholds to predict recurrence. In the study by Ascione et al. [43], the proposed cutoffs for synovial fluid leukocyte and neutrophil percentage were found to outperform those proposed by other authors (Table 3) [46].

**Table 3:** Investigation on proposed thresholds for leukocyte count and relative neutrophils percentage

Author (reference)	Population	Proposed cutoff	Sensitivity	Specificity
Kusuma SK [44]	76 knee spacers	1102 cells/µL 71% PMN	75% 75%	61% 66%
Hoell et al. 2016 [46]	115 spacers (56 hips and 59 knees)	970 cells/µL NA	31% NA	39% NA
Newman et al. 2017 [41]	77 hip spacers	3000 cells/µL 80% PMN	47% 76%	87% 80%
Zmistowski et al. 2017 [42]	128 spacers (40 hips and 88 knees)	1234 cells/µL 57% PMN	44% 67%	77% 59%
Boelch et al. 2018 [45]	94 knee spacers	4450 cells/uL NA	50% NA	76% NA
Ascione et al. 2021 [43]	82 knee spacers	934 cells/µL 52%	82% 82%	82% 78%

*NA* not available, *PMN* polymorphonuclear leukocyte

Alpha-defensin can be considered a promising synovial fluid biomarker for detecting PJI because it is released in the synovial fluid by the polymorphonuclear cells in response to bacterial pathogens. However, the predictive value of synovial fluid a-defensin has shown conflicting results, likely due to different techniques used in investigations. A meta-analysis of 13 studies on PJI patients indicated that both enzyme-linked immunosorbent assay (ELISA) and lateral flow tests can effectively detect a-defensin in synovial fluid specimens, demonstrating favourable sensitivity and specificity for ruling out PJI and identifying aseptic prosthetic joint loosening, but the precise role of synovial fluid a-defensin at the time of reimplantation was brought into question [47]. A retrospective multicenter study found that a-defensin has poor efficacy in ruling out persistent infection prior to reimplantation in 14 out of 69 patients investigated [48]. These results are in line with those described by Stone et al. [49] with regard to 46 patients who reported a treatment failure rate of nearly 20% despite reimplantation that was performed with a negative a-defensin test. Similar results were reported by Owen et al. [50] with regard to a cohort of 87 patients undergoing resection arthroplasty for PJI. The accumulated data suggest that the routine use of a-defensin may not be warranted, as it does not distinguish infected patients from those potentially having infection during the second stage of a two-stage exchange.

## Upcoming evidence in two-stage revision arthroplasty: an ideal scoring system

As no single test has a sufficient accuracy and specificity to definitively rule out persistent infection, a diagnostic score that incorporates the results of significant investigations routinely performed at the time of reimplantation could be valuable for identifying patients at the highest risk of recurrence.

Recently, a scoring system has been developed by analysing numerous blood and synovial fluid parameters routinely obtained at the time of reimplantation. This scoring system is intended for patients on continuous antibiotic therapy who undergo reimplantation without clinical signs or symptoms of infection and inflammatory indices within the normality range or significantly downloading [51, 52]. After conducting multivariate analysis, D-dimer levels > 1100 µg/mL, synovial cell counts > 934/mL and PMN percentages > 52% were identified to be independently linked with an unfavourable outcome. The b-score derived from multivariate analysis for each parameter was calculated and rounded to generate the final diagnostic score to be used before reimplantation, as reported in Table 4. A higher score indicates a greater risk of PJI recurrence following reimplantation. The practical implication of this score is that patients with a score exceeding 2 should not undergo reimplantation but instead should be considered for repeating debridement and spacer exchange due to their high risk of persistent or recurrent PJI. However, patients with a score of 2 or lower can proceed with definitive reimplantation with the lowest risk of recurrence [52].

**Table 4:** A proposed scoring system predicting infection recurrence

Proposed criteria	Threshold	Score	Decision
Serum D-dimer (ng/mL)	1110	1.5	Score
Synovial WBC (cells/µL)	934	2	≤ 2 = low risk of recurrence of infection
Synovial PMN (%)	52	2	> 2 = high risk of recurrence of infection

**Fig. 1:**
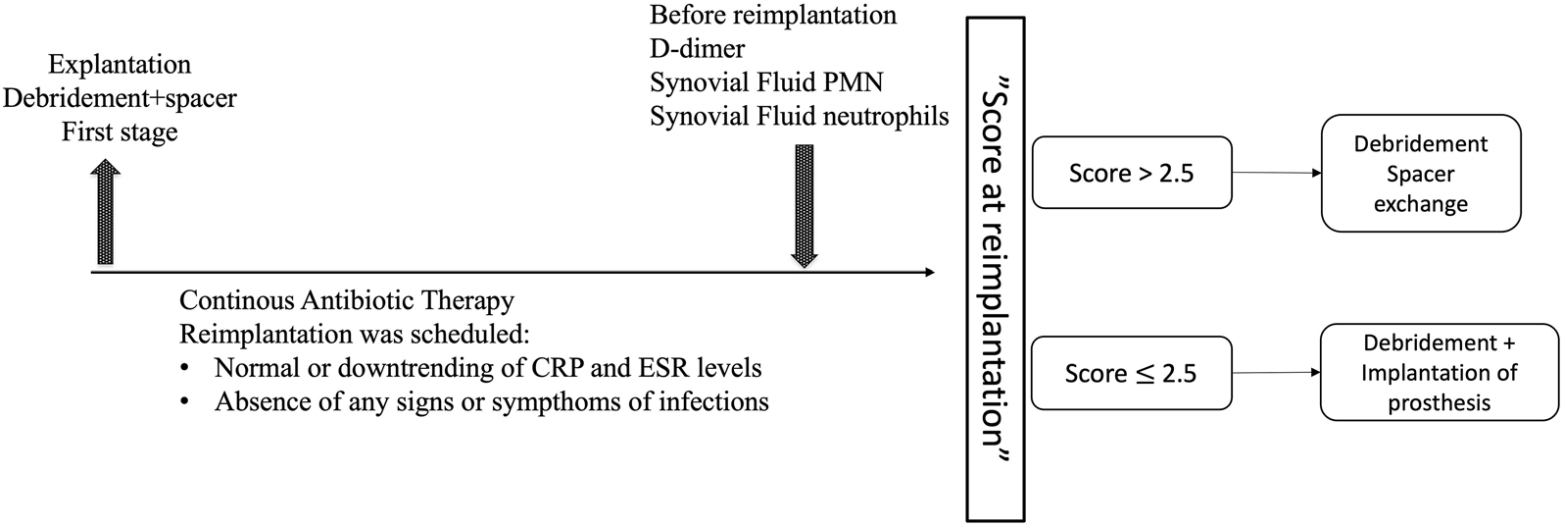
A proposed treatment algorithm for patients undergoing two-stage exchange after prosthetic joint infection. i.v., intravenous

Considering all these findings, this scoring system allows surgeons to establish a new treatment algorithm (Fig. 1). Following the first stage, patients undergo reimplantation while still on antibiotic therapy. Reimplantation can be scheduled for patients meeting specific criteria, including the absence of local signs or symptoms of infection as well as CRP levels and ESR that are normal or downloading. At least 1 week before the second stage, serum D-dimer, synovial fluid leukocyte count and neutrophil percentage should be measured to finalize the score and evaluate the risk of recurrence. The second stage of revision surgery should be planned in patients with a diagnostic score of 2 or below.

## Conclusions

Successful management of patients with PJI undergoing a two-stage exchange requires a multidisciplinary approach. Orthopaedic surgeons need to collaborate as part of a team that includes infectious diseases specialists, microbiologists, primary care physicians and relevant professionals such as nutritionists, psychiatrists and plastic surgeons. This collaborative approach allows for optimization of the patient’s preoperative condition, which can positively affect surgical outcomes and postoperative recovery.

Although two-stage exchange should be considered the best treatment for patients experiencing a PJI, this procedure still reports a significant failure rate, mainly due to the infection recurrence after definitive reimplantation. Identifying the cases at high risk of infection recurrence would be a significant improvement in terms of disability time reduction, relative expenditures and rate of patients experiencing definitive disability [39].

Many studies have focused on the investigations proposed to diagnose PJI, but literature results are frequently inconsistent and do not effectively address this complication, potentially leading to poor outcomes. Only a few studies have been planned to establish which investigation can report a significant sensibility or susceptibility to be routinely employed to support the choice of reimplantation in those undergoing two-stage replacement. In fact, ERS and CRP have an important role in diagnosing PJI, but their value in assessing infection cure at the time of reimplantation has been brought into question, as the relationship between a negative value of these biomarkers and microbiologic eradication is controversial, particularly when the strategy associated with the highest success rate (continuous therapy strategy) is adopted [53]. Moreover, microbiologic investigations at the time of reimplantation cannot be considered effective in diagnosing or excluding infection persistence, and their value should be contextualized [54].

The most attractive investigations can be performed on synovial fluid, and both leukocyte count and neutrophil percentage have demonstrated a high predictive value. In this contest, Ascione et al. identified different thresholds from those adopted at the time of PJI diagnosis that showed a favourable sensibility and specificity to sustain PJI cure and identified D-dimer as an attractive biomarker. Based on these findings, a new diagnostic score demonstrated a favourable predictive role in identifying the patients at the highest risk of failure after definitive reimplantation, despite other clinical examinations and laboratory investigations not supporting infection persistence [52]. Its use could be effective in identifying patients with infection persistence needing further procedure and antibiotic treatment prior to definitive reimplantation.

## Data Availability

Not applicable to a narrative review.
